# Effect of Parameters on Oxychlorination of *Tert*-Butyl Ethers

**DOI:** 10.1080/15376510701623615

**Published:** 2008-06-23

**Authors:** Jerzy Gaca, Alicja Gackowska, Natalia Belt

**Affiliations:** Department of Chemistry and Environmental Protection, Faculty of Chemical Technology and Engineering, University of Technology and Agriculture, Seminaryjna 385-326, Bydgoszcz, Poland

**Keywords:** Chlorocompounds, Effect of Molar Ratios, pH, Temperature, *Tert*-Butyl Ethers

## Abstract

The effect of concentration, molar ratios of reagents, pH, and temperature on formation of chloro-organic products in reaction of *tert*-butyl ethers with chloride ions and hydrogen peroxide has been determined. A significant effect of Cl^−^ ions and H_2_O_2_ molar ratios on the rate of chloro-organic product formation has been observed. Studies on oxychlorination of *tert*-butylethyl ether (ETBE) at pH 7, 3.5, and 2.5 have been carried out. It was found that introduction of hydronium ions into the reaction system considerably hastened the process of chloro-organic product formation. Hydronium ions contribute to the formation of the reactive tert-butyl carbocation, which undergoes secondary reactions in the presence of reactive forms of chlorine and oxygen. Moreover, the effect of temperature on ETBE (*tert*-butylethyl ether) and MTBE (*tert*-butylmethyl ether) conversions was verified. The reactions of MTBE and ETBE oxychlorination were carried out at temperatures of 5°C, 20°C, and 35°C.

## INTRODUCTION

*Tert*-butyl ethers are added to fuels as the oxygen components since they increase the octane number of petrol. They contribute to the improvement of air quality by reducing the emission of carbon oxide and precursors of free radicals in the exhaust gases. On the other hand, out-of-control leakages of fuels containing *tert*-butyl ethers are the source of their presence in the environment. Ethers are identified in ground, surface, storm, and waste waters as well as in air (Squillace et al. 1996; EPA 1998; Halden et al. 2001). Squillace et al. (1996) showed that MTBE is the second, after chloroform, volatile organic compound most often identified in ground waters. MTBE concentration in rain water as well as in ground water is significantly conditioned by the season of the year. It was shown that concentration of ethers in the water environment was higher in winter than in summer time (Delzer et al. 1996; Achten et al. 2001; Baehr et al. 2001). The available data suggest that the volatile organic compounds are characterized by higher stability at low temperatures (below 10°C) than at higher ones. Temperature rise contributes to their quick vaporization and increase of their chemical activity in the gaseous phase. An increase in the amounts of the volatile organic compounds in air enhances the probability of their contact with radical forms and oxidative agents. Moreover, ethers being cumulated in air during the rainy season are washed out and they migrate together with rain waters to ground waters. This fact is of particular importance in urban areas because of the frequent traffic jams and numerous petrol stations that are potential sources of ether emission into the environment. On the other hand, chloride ions, oxidants, and pH-reducing agents present in waters can result in conversion of ethers to compounds that are much more aggressive for the environment.

Our studies showed that *tert*-butyl ethers in the presence of oxidative agents and chloride ions can be a source of toxic chloro-organic compounds (Gaca et al. 2004; Gackowska and Gaca 2004; Cysewski et al. 2006). As a result of reaction of MTBE/or ETBE with HCl/H_2_O_2_ and metal chloride/oxidant/H^+^ system, we obtained 2-chloro-2-methylpropane, 3-chloro-2-methylpropene, 1,2-dichloro-2-methylopropane, 3-chloro-2-chloromethylpropene, and chloroacetone besides products of oxidation such as *tert*-butyl alcohol, acetone, 2-methylpropene, and *tert*-butyl hydroperoxide. The mechanism of formation of the identified dichlorocompounds was described by Cysewski et al. (2006).

In order to check which factors have an effect on the rate of chloro-organic compound formation, studies on the effect of concentration, molar ratios of reagents, pH, and temperature were undertaken. On the basis of investigations carried out by O'Reilly et al. (2001), Achten et al. (2002), Arp and Schmidt (2004), and Fischer et al. (2004), it can be assumed that pH and temperature will play a significant role in the process of chloro-organic compound formation since the above-mentioned parameters have an effect on the stability of *tert*-butyl ethers. We can assume that different behaviors of ethers at various ambient temperatures and at various pH values will affect the formation of ether conversion products.

## EXPERIMENTAL

MTBE 98%, 1,2-dichloro-2-methylpropane, 3-chloro-2-chloromethylpropene (Sigma-Aldrich Germany), ETBE (Polish Petrol Concern Orlen” S.A. Płock Poland), *tert*-butyl alcohol, hydrochloric acid 36%, nitric acid (V) 65%, hydrogen peroxide 30%, and sodium chloride (Polish Chemical Reagents) were used in our studies. Samples for experiments were prepared by liquid-liquid extraction technique. Carbon disulfide (Merck) was applied as a solvent. Samples were analyzed by gas chromatograph HP 5890-Hewlett Packard equipped with flame ionization detector FID. Operating parameters were as follows: column HP-1 (0.53 mm × 60 m × 0.2 μm) temperature of injector 250°C, temperature of detector 250°C, temperature program: 40°C/4 min-10°C/min-200°C.

Precision was evaluated by injection of samples (repeated six times) obtained after extraction of ETBE, 1,2-dichloro-2-methylpropen, and 3-chloro-2-chloromethylpropene solutions at concentrations of 1 mL/L, 10 μL/L, and 10 μL/L, respectively. The values of relative standard deviations were determined as follows: ETBE, 3.37%; 1,2-dichloro-2-methylpropen, 2.84%; and 3-chloro-2-chloromethylpropene, 4.41%.

In the next stage, a linearity of calibration curves for ETBE 1,2-dichloro-2-methylpropan and 3-chloro-2-chloromethylpropene was determined. For that purpose, water solutions of ETBE (10, 5, 1, 0.5, 0.2, and 0.05 mL/L) and 1,2-dichloro-2-methylpropen and 3-chloro-2-chloromethylpropene (20, 10, 5, 1, and 0.5 μL/L) were prepared. Then, liquid-liquid extraction was performed by the use of carbon dioxide and calibration curves were determined. The linear correlation coefficients (R^2^) were as follows: ETBE, 0.987; 1,2-dichloro-2-methylpropan, 0.994; and 3-chloro-2-chloromethylpropene, 0.996.

ETBE solution at a concentration of 0.07 mol/L with the addition of 0.7 mol of NaCl and 0.35 mol of H_2_O_2_ was applied in our investigations. In order to determine the effect of pH on the process of chloro-organic product formation, the samples with pH values of 7, 3.5, and 2.5 were prepared. Solutions at pH values of 2.5 and 3.5 were obtained by addition of suitable amounts of nitric acid (V). pH of solution was measured by pH-meter Elmetron CX-741.

Studies on the effect of temperature were carried out in the presence of ETBE and MTBE as well as *tert*-butyl alcohol (TBA), which was the product of oxidation of ethers. Composition of the solution studied is presented in Table 1.

**TABLE 1:** Composition of the solutions studied

	ETBE/or MTBE/or TBA		NaCl/or HCl		H_2_O_2_		HNO_3_	
				
No. of sample	mol	mL	mol		mol	mL	mol	mL
4^a^	0.07	9.6	0.7	40.09*g*	0.35	36.0	0.07	48.5
5^b^	0.07	9.6	0.7	62 mL	0.35	36.0	—	—
6^a^^,^^c^	0.07	8.3	0.7	40.09*g*	0.35	36.0	0.07	48.5
7^b^^,^^c^	0.07	8.3	0.7	62.0*mL*	0.35	36.0	—	—
8^a^^,^^d^	0.07	5.2*g*	0.7	62.0*mL*	0.35	36.0	—	—

a With NaCl.

b With HCl.

c With MTBE.

d With TBA.

## RESULTS AND DISCUSSION

The degradation rate of ether and formation of 1,2-dichloro-2-methylpropane, 3-chloro-2-chloromethylpropene, and TBA depend on molar rations of reagents, concentration of hydronium ions, and temperature. Molar ratios of reagents are of the fundamental importance. By applying the excess of hydrogen peroxide and hydrochloric acid in relation to ETBE, the process of chlorocompound formation was hastened (Gaca et al. 2004). It can be explained by the specific character of the H_2_O_2_/Cl^−^/H^+^ system. According to the literature data, the H_2_O_2_/Cl^−^/H^+^ system is of the complex character and it is described by a series of secondary reactions. They are both ionic (Livingstone and Bray 1925; de la Mare et al. 1954) and radical (Davies and Kustin 1973) reactions. In both cases, the reactive forms of chlorine (RFCh) are formed. The ions Cl^−^, ClO^−^ ClO_2_^−^, and Cl^+^ are formed by ionic path, whereas Cl^·^ radical is formed by radical reactions. The formed RFCh can further react with chloro-organic compounds present in solution with formation of chloro-organic products. It results from the above statement that neither the ionic nor the radical path in the process of chloro-organic product formation by reaction of *tert*-butyl ethers with H_2_O_2_/Cl^−^/H^+^ system can be excluded.

**FIGURE 1:**
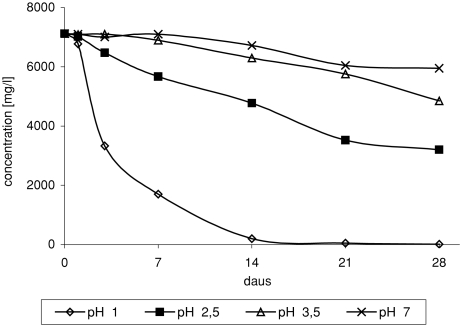
Change of ETBE concentration in time depending on pH, in reaction of ETBE with NaCl/H_2_O_2_/HNO_3_.

It was observed that H^+^ ions play an important role in the process of toxic chloro-organic compound formation. In neutral medium, ether was relatively stable. No chloro-organic derivatives were identified in the products. Insignificant ETBE loss in reactions proceeding with participation of hydrogen peroxide and sodium chloride at pH 7 proves to slow the process of ether oxidation (Fig. 1).

**FIGURE 2:**
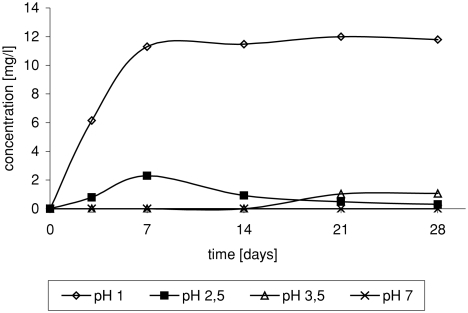
Change of 1,2-dichloro-2-methylopropane concentration in time depending on pH, in reaction of ETBE with NaCl/H_2_O_2_/HNO_3_.

Acidification of the medium resulted in series of reactions leading to formation of chloro-organic compounds as well as the products of ether oxidation and degradation (Gaca et al. 2004). In the case of 1,2-dichloro-2-methylpropane, it was observed that in solution at pH 3.5, small amounts of product were formed after a long time. Reduction of the pH value resulted in shortening of time of product formation. In solution at pH 1, product was formed relatively quickly and in higher amounts (Fig. 2).

**FIGURE 3:**
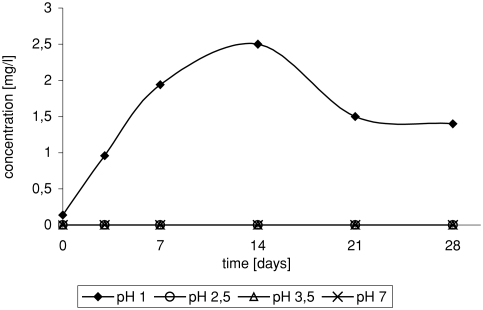
Change of 3-chloro-2-chloromethylopropene concentration in time depending on pH, in reaction of ETBE with NaCl/H_2_O_2_/HNO_3_.

Strongly acid medium is required in the process of 3-chloro-2-chloromethylpropene formation (Fig. 3). The presence of 3-chloro-2-chloromethylpropene was observed only in solution at pH 1.

**SCHEME 1:**
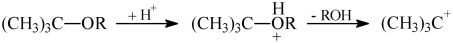
Reaction of *tert*-butyl ether in the presence of H^+^ ions.

Formation of *tert*-butyl ether conversion products can be explained by the fact that in the first stage of reaction, dealkoxylation and carbocation formation occur under the influence of H^+^ ions present in the reaction mixture (Scheme 1).

The formed carbocation can further react and in the first stage, *tert*-butyl alcohol, 2-chloro-2-methylpropane, and 2-methylpropene are formed. Then, as a result of the primary product conversions, 1,2-dichloro-2-methylpropane and 3-chloro-2-chloromethylpropene are formed (Cysewski et al. 2006).

**FIGURE 4:**
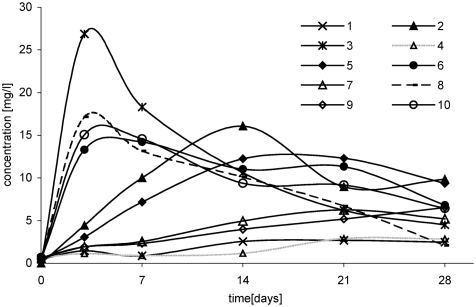
Change of 1,2-dichloro-2-methylpropane concentration in time, depending on temperature, in following reactions: ETBE/NaCl/H_2_O_2_/HNO_3_: 1, (5°C); 2, (20°C); 3, (35°C), ETBE/HCl/H_2_O_2_: 4, (5°C); 5, (20°C); 6, (35°C), MTBE/NaCl/H_2_O_2_/HNO_3_: 7, (20°C); 8, (35°C), MTBE/HCl/H_2_O_2_: 9, (20°C); 10, (35°C).

The successive parameter that has an effect on oxychlorination of *tert*-butyl ethers is temperature. Studies showed that at a temperature of 5°C, the increase in 1,2-dichloro-2-methylpropane was insignificant and it proceeded very slowly both in reaction of ETBE with NaCl/H_2_O_2_/HNO_3_ and of ETBE with HCl/H_2_O_2_ (Fig. 4, series 1 and 4). Temperature rise by 15° contributed to the increase in chlorocompound yield and the rate of its formation (Fig. 4, series 2 and 5).

In reaction with participation of MTBE, the effect of temperature on 1,2-dichloro-2-methylpropane formation is similar. However, it is formed more slowly and with the lower yield (Fig. 4, series 7 and 9).

The higher concentration of chloro-organic product was obtained in the case when the temperature of the reaction system was 35°C. After 3 days, in reactions of ETBE with NaCl/H_2_O_2_/HNO_3_ and with HCl/H_2_O_2_, concentration of 1,2-dichloro-2-methylpropane was 27 mg/L and 13 mg/L (Fig. 4, series 3 and 6); however, in reactions with MTBE, it was 17 mg/L and 15 mg/L, respectively (Fig. 4, series 8 and 10).

In reactions with participation of sodium chloride (Fig. 4, series 3 and 8), concentration of 1,2-dichloro-2-methylpropane was higher than that in reactions with HCl (Fig. 4, series 6 and 10).

In reactions of both ethers (ETBE and MTBE) with hydrochloric acid and hydrogen peroxide at a temperature of 35°C, processes of chloroproduct formation and decomposition proceeded similarly.

**FIGURE 5:**
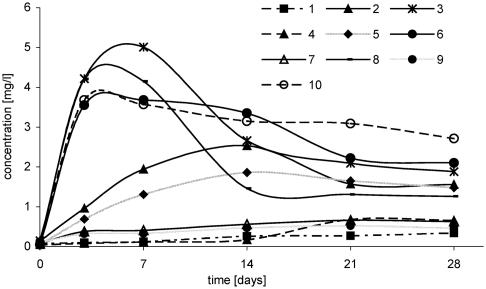
Change of 3-chloro-2-chloromethylpropene concentration in time, depending on temperature, in the following reactions: ETBE/NaCl/H_2_O_2_/HNO_3_: 1, (5°C); 2, (20°C); 3, (35°C), ETBE/HCl/H_2_O_2_: 4, (5°C); 5, (20°C); 6, (35°C), MTBE/NaCl/H_2_O_2_/HNO_3_: 7, (20°C); 8, (35°C), MTBE/HCl/H_2_O_2_: 9, (20°C); 10, (35°C).

Similar effect of temperature was observed during formation the second chloroproduct (i.e., 3-chloro-2-chloromethylpropene). At a temperature of 5°C, 3-chloro-2-chloromethylpropene was formed very slowly and in small quantities (Fig. 5, series 1 and 4). At a temperature of 35°C, 3-chloro-2-chloromethylpropene was formed in the highest concentration (about 5 mg/L) after 7 days of reaction between ETBE and NaCl/H_2_O_2_/HNO_3_ (Fig. 5, series 3). However, in reaction of ETBE with HCl/H_2_O_2_, the yield of dichlorocompound was lower and at a temperature of 35°C, its concentration was about 4 mg/L (Fig. 5, series 6). Similar results were obtained in reaction with participation of MTBE (Fig. 5, series 8 and 10). However, at a temperature of 20°C, 3-chloro-2-chloromethylpropene was formed in higher quantities in reaction with ETBE than in reaction with MTBE (Fig. 5, series 2 and 7, 5 and 9).

**FIGURE 6:**
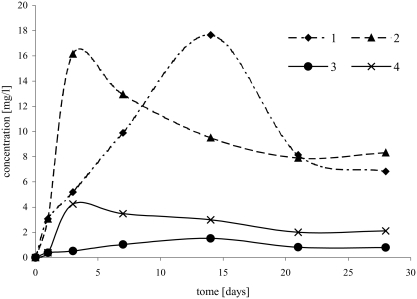
Change of dichlorocompound concentrations in time, depending on temperature, in reaction of TBA with HCl/H_2_O_2_: 1,2-dichloro-2-methylpropane: 1, (20°C); 2, (35°C), 3-chloro-2-chloromethylpropene: 3, (20°C); 4, (35°C)

Moreover, reactions of TBA with the H_2_O_2_/HCl system at temperatures of 20 and 35°C were carried out. At higher temperature, the reaction runs at higher rate (Fig. 6).

The results described above are consistent with those presented by Huang et al. (2002), who showed that in the process of MTBE oxidation in the presence of persulfates (without chloride ions) at temperatures within the range of 20°C to 50°C, the highest amounts of ether were converted at a temperature of 50°C. Studies on the effect of temperature showed also that ETBE in the presence of Cl^−^, H^+^ ions, and hydrogen peroxide was more reactive than MTBE. The similar effect of reactivity of ethers on their conversions was observed by Norris and Rigby (1932), who showed that *tert*-butyl chloride was formed more rapidly in reaction of 27% HCl with ETBE than with MTBE.

## CONCLUSION

The stables in neutral medium ethers can migrate to considerable distances and thus, they contribute to contamination of drinking waters. In the case when they come into contact with acid sewage, they can contribute to formation of new, more reactive products, which, in turn, in the presence of reactive forms of chlorine, are a potential source of toxic chloro-organic products. On the basis of results obtained, it can be assumed that intensity of chloro-organic compound formation is higher in summer than in the winter season. Therefore, introduction of *tert*-butyl ethers into the environment has the repercussions in formation (as a result of the secondary reactions) of the successive portion of chloro-organic compounds that are relatively stable and can cumulate in various organisms.
